# Reconstruction of the skull base in spontaneous rhinoliquorrhea

**DOI:** 10.3205/iprs000137

**Published:** 2019-07-16

**Authors:** Mark Jakob, Mattis Bertlich, Klaus W. Eichhorn, Marcus Thudium, Friedrich Bootz, Thorsten Send

**Affiliations:** 1 Department of Otorhinolaryngology, Head and Neck Surgery, University Hospital Munich, Germany; 2 Otorhinolaryngology, Head and Neck Surgery, University Hospital Bonn, Germany; 3 Department of Anesthesiology, University Hospital Bonn, Germany

**Keywords:** endonasal surgery, rhinoliquorrhea, spontaneous, rhinoliquorrhea

## Abstract

**Objective/Hypothesis:** Spontaneous rhinoliquorrhea often occurs due to defects of the skull base. It is often misinterpreted as rhinitis and is surgically the most difficult rhinoliquorrhea entity to close.

**Methods:** We conducted a retrospective chart analysis of patients that were diagnosed with spontaneous rhinoliquorrhea at the University Hospital Bonn between 2001 and 2017.

**Results:** Overall, twelve patients were included in this study. On average, the time between occurrence of nasal discharge and diagnoses of rhinoliquorrhea was 123 days. In ten patients, the localization of the skull base defect could be localized by computed tomography or MRI cisternography. Ten patients underwent surgery, of which 9 remained recurrence free. One patient underwent revision surgery and from thereon was recurrence free.

**Conclusion:** Spontaneous rhinoliquorrhea still remains a diagnostic and therapeutic challenge. Whenever persistent watery nasal discharge appears in a patient, rhinoliquorrhea must be considered. Endoscopic surgical reconstruction of the skull base is the therapeutic gold standard and should be attempted as soon as the diagnosis is secured.

## Zusammenfassung

**Hypothese:** Rhinoliqurrhoe tritt häufig als Folge von spontanen Schädelbasisläsionen auf; diese Form der Rhinoliqurrhoe ist die am schwierigsten zu beherrschende.

**Methoden:** Wir haben eine retrospektive Analyse aller Patienten durchgeführt, welche mit der Diagnose einer spontanen Rhinoliquorrhoe im Uniklinikum Bonn behandelt wurden.

**Ergebnisse:** Insgesamt wurden zwölf Patienten in dieser Studie eingeschlossen. Im Mittel betrug die Zeit zwischen dem ersten Auftreten einer wässerigen Rhinorrhoe und der Diagnose einer spontanen Rhinoliqurrhoe 123 Tage. In zehn dieser Patienten konnte der Defekt mittel Computertomographie oder MR-Zisternographie dargestellt werden. Zehn Patienten wurden aufgrund der spontanen Rhinoliqurrhoe operiert, von denen neun auch im weiteren Verlauf beschwerdefrei blieben. Ein Patient benötigt einen Revisionseingriff, war hernach jedoch ebenfalls ohne weitere Episoden von Rhinoliqurrhoe.

**Schlussfolgerung:** Spontane Rhinoliqurrhoe ist nach wie vor sowohl diagnostisch wie therapeutisch eine Herausforderung. Bei jedem Patienten, der sich mit wässriger Rhinorrhoe vorstellt, muss eine spontane Rhinoliqurrhoe in Betracht gezogen werden. Die endoskopische Rekonstruktion der Schädelbasis ist bis heute der therapeutische Goldstandard und sollte bei Diagnosestellung geplant werden.

## Introduction

The term rhinoliquorrhea is a clinical feature that describes the phenomenon when cerebrospinal fluid (CSF) drains from the skull base through the paranasal sinuses, presenting as rhinorrhea and indicating a physical breach of the integrity of the subarachnoid space.

The most common reason for rhinoliquorrhea is a previous trauma [1], but also iatrogenic manipulations are considered to be common origin of CSF rhinorrhea [1], [2], [3]. Apart from those origins, rhinoliquorrhea may also occur spontaneously. The incidence of spontaneous rhinoliquorrhea varies in literature; it ranges from approximately 3% [4] up to almost 50% [1].

Common risk factors for spontaneous rhinoliquorrhea are female gender, middle age and overweight [5], [6], [7]. In addition to this, elevated intracranial pressure (ICP) has been discussed as an additional risk factors for spontaneous CSF rhinorrhea [8], [9]. It is believed that elevated ICP leads to a thinning of the skull base, eventually leading to minor traumas like increases in abdominal pressure to cause CSF leaks [9].

Finally, a persistent cranipharyngeal duct in the lateral recess of the sphenoid sinus has been proposed to also pose a risk factor for spontaneous rhinoliquorrhea [10]. However, there has been a considerable debate about this etiology [11], [12] and the clinical importance is probably limited to few individual cases [13].

Undetected and untreated rhinoliquorrhea poses a considerable risk for the patient, since the origin of the liquorrhea may also act as an entryway for serious infections of the central nervous system like meningitis, encephalitis or even intracranial abscess formation. Since spontaneous rhinoliquorrhea has no distinct starting event like a trauma, it is often misdiagnosed for a considerable amount of time, increasing the risk of grave complications [1]. In addition to this, spontaneous rhinoliquorrhea is known to have the highest recurrence rates of all types of rhinoliquorrhea, making it the surgically most challenging entity [14], [15].

We therefore conducted a retrospective analysis of all cases of spontaneous rhinoliquorrhea that presented at the University Hospital of Bonn with particular respect to the diagnosis and the surgical reconstruction of the skull base.

## Patients and methods

All patients that were registered with the ICD-10 code for extrusion of cerebrospinal liquor and that had been treated in the ENT department between 2001 and 2017 were included in this study. Patients that had a previous history of skull base or paranasal sinus surgery or a relevant trauma to the head and neck region as well as malignancies arroding the skull base or other organic reasons for CSF rhinorrhea were excluded from the study.

The patients details were taken from the electronical patient file as well as the doctoral records and from the nursing staffs files. Details included age, gender, date of presentation, risk factors, duration of discharge from the nose, other initial symptoms, potential previous treatment of the symptoms as well as imaging modalities used to locate the skull base defect. 

When it came to treating the skull base defect, we recorded the type of surgery that was performed as well as the surgical techniques and materials that were used to cover the defect. We also recorded the follow-up of the success of the operation and potential complications after the surgery.

Overall, 12 patients met the inclusion criteria and were included in this study.

## Results

### Study population

Overall, twelve patients were included in the study. Out of these, 11 (87.5%) were females. The median age upon presentation was 44.3±15.1 years.

### Symptoms upon presentation

Out of the patients included in the study, 1 patient had underweight upon presentation, 1 patient had regular weight and 3 patients presented with overweight. Six patients showed obesity upon presentation. For the other patient, the patient chart did not supply any weight data (Table 1 (Tab. 1)).

All patients presented with a clear discharge from the nose; additionally, one patient showed recurring syncopes and vertigo, one patient presented with photophobia and one patient presented with cephalgia. Three patient presented with a history of meningitis or clinical suspicion of meningitis. Three patients had previously received treatment for suspicion of vasomotor or allergic rhinitis. On average, clear discharge from the nose had been present for 123.9±198.7 days. 

### Diagnostics

Upon the suspicion of nasal rhinoliquorrhea, all patients had samples of their nasal discharge collected and sent to the laboratory for further testing. Determination of β-trace protein was done in ten out of the twelve patients; positive results were yielded in eight cases (80%). In two more patients, glucose and overall protein levels were measured in the nasal discharge; these were positive in both cases. 

**Figure 1:**
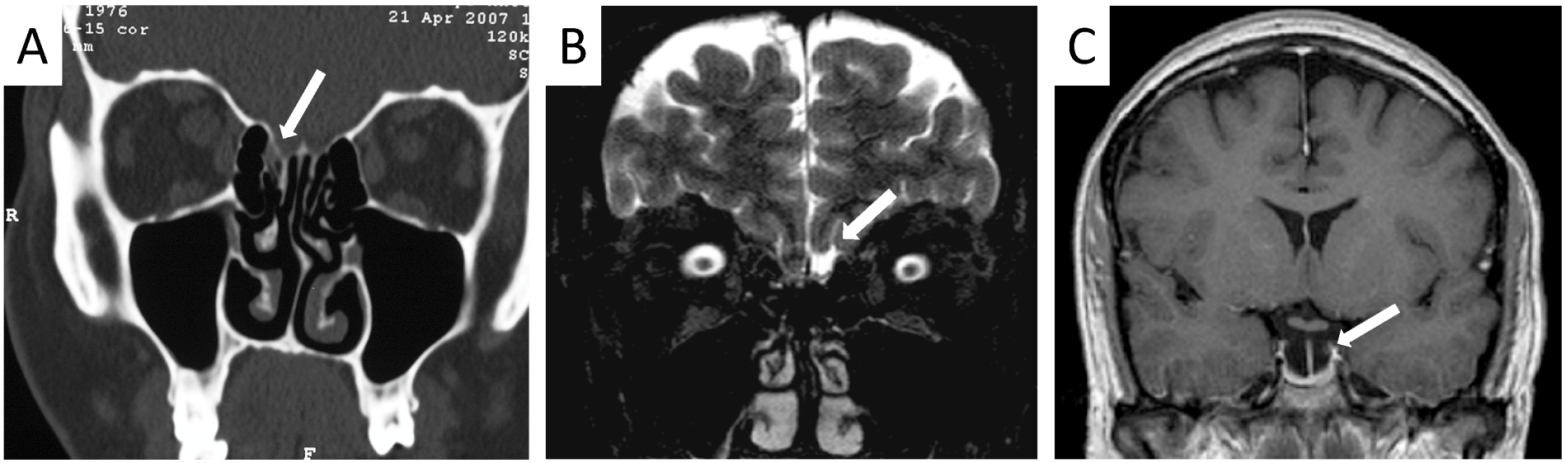
A) CT: CSF-leak of the lamina cribrosa right side. B) MRI: CSF-leak of the lamina cribrosa left side. C) MRI: empty sella syndrome.

Patients that were diagnosed with nasal rhinoliquorrhea had either already received or immediately received high resolution computed tomography of the skull base and the paranasal sinuses. In five cases (41.6%), a lesion could be identified in the computed tomography (Figure 1 (Fig. 1), Table 2 (Tab. 2)). Furthermore, eight patients received magnet resonance imaging (MRI) with intrathecal application of contrast material (Figure 1B (Fig. 1); MRIFF cisternography). Out of these eight patients, a lesion could be identified in six cases (75%). In two patients, a meningocele (16.7%) and in one patient (8.3%), an empty sella syndrome was found.

### Surgery

Upon diagnosis of nasal rhinoliquorrhea, surgical reconstruction of the skull base was recommended to all patients. Out of the twelve patients included in this study, ten agreed to surgical reconstruction of the skull base to stop rhinoliquorrhea (Table 3 (Tab. 3)). In eight of those patients, a skull base defect with rhinoliquorrhea could be found. The surgical approach was nine times entirely endoscopic and once a combination of an endoscopic and transcranial approach. Grafts used were five times fascia lata, twice abdominal fat and once alloplastic material, ear cartilage or a combination of fascia lata and ear cartilage, respectively.

### Follow-up of patients after surgery

All patients recovered quickly after surgery. Median follow-up was 7.7±11.5 months. Out of the patients were surgery was performed, one patient presented with recurrent nasal rhinoliquorrhea; the other patients did not present with recurrent rhinoliquorrhea. No severe adverse effects of the surgery were seen in the patients.

## Discussion

Diagnosis of spontaneous rhinoliquorrhea still remains a challenge for any clinician to this day – this is highlighted by the fact that patients had been suffering from clear nasal discharge for an average of over four months. However, a quick diagnosis is crucial in adequate treatment of spontaneous rhinoliquorrhea, as is highlighted by the fact that three patients had a history of meningitis, a potentially life-threatening and often debilitating disease, before the diagnosis of spontaneous rhinoliquorrhea had been confirmed. 

Rhinoliquorrhea should especially be considered if the patient is female, above the age of forty and has an increased body-mass index as highlighted by our patient data [7].

We were able to show that β-trace is a relatively specific marker for rhinoliquorrhea; hence we would strongly recommend β-trace analysis of the discharge in any patient presenting with persistent and clear nasal discharge over the course of more than a week. This recommendation is in line with previous studies addressing this issue [14], [16].

Once a nasal cerebrospinal fluid leak has been confirmed, high resolution computed tomography should be done in order to localize the skull base defect. In our collective, this was only possible in 50% of the cases. However, it has been reported that high resolution computed tomography is regularly inaccurate in predicting the site of a nasal CSF leak in approximately 25% of cases [17].

Hence, additional diagnostics are necessary if the leak cannot be identified by high resolution computed tomography alone. In our patient collective, MRI cisternography proved effective in 75% of cases in localizing the site of the CSF leak. Thus, we would recommend MRI cisternography if computed tomography is not available or its results are ambiguous [18]. In addition to this, we have demonstrated that MRI imagining of the head may reveal additional findings like an empty sella syndrome or a meningoencephalocele which are both associated with an increased risk for rhinoliquorrhea [19], [20], [21] and may even pose a surgical risk.

If location by these methods alone is not possible, perioperative intrathecal application of fluoresceine [22] may be considered.

However, once rhinoliquorrhea is confirmed, surgical reconstruction of the skull base is the primary treatment option that should be considered. The gold standard is the endonasal endoscopic approach. Mostly it avoids the typical collateral damage in approaches like the frontal craniotomy and offers an unmatched visualization of the defect. Finally, the endoscopic approach offers high success rates as has been shown by our collective; fittingly, these results are in line with recent literature addressing this topic [23]. 

The types of grafts that are then used to close the defect are numerous and include fascia lata, abdominal fat, various mucous membrane flaps and alloplastic materials [24]. However, there seems to be no impact on the outcome of the surgery which graft material is used [25]. 

When it comes to the reconstruction of the skull base and closure of the defect, underlay and only techniques have been described [26]. There is some debate as to whether the onlay or underlay approach yields the best results, a recent retrospective analysis has suggested that a combination of both these techniques show the highest recurrence free closure rate in the ethmoid roof and the sphenoid sinus [22]. 

A nasoseptal flap may also be considered for larger defects in these regions [27]; however, it has limited reach, underlay techniques are difficult to perform with this flap and it may cause anosmia if larger areas of the ethmoid roof are covered by it. Another special case are CSF leaks situated in the olfactory grove and the cribriform plate; these are usually hard to access for an underlay technique and inconsiderate manipulation may potentially widen the defect; therefore, onlay techniques should be the main choice here. If there is a defect in the lateral sphenoid sinus, good exposition of the defect is often difficult and a combined approach by both ENT- and neurosurgeons through the pterygoid may be considered [28]. However, since each patients anatomy may considerably and will probably differ from the norm, fair preoperative imagining and subtle preoperative planning is paramount for successful skull base reconstruction.

As to whether a lumbar drain should be placed in order to decrease intracranial pressure and therefore aid in the healing process of the skull base defect, there is considerable debate addressing this topic. While some authors favor this approach [29], most authors have come to agree that there is no benefit in a long term lumbar drainage [30], [31]. In addition to its ambiguous role in skull base reconstruction, the complication a long term lumbar drainage may pose are actually quite severe, like pneumencephalon [22]. Hence, we would not recommend a lumbar drain.

It has been reported in the past that endoscopic reconstruction of the skull base in spontaneous rhinoliquorrhea yields high success rates of up to 90% and that revision surgery if often successful [32]. In our patient cohort, we were able to close the defect in the first attempt in 90% and in 100% on the second attempt. In this respect, our results are in line with the literature. However, it has to be kept in mind that the collective at hand is a relatively small one; recurrence rates of up to 25% and over a period of several months have been reported [33]. Therefore, there should be regular and long-term follow-up to detect recurrences early and avoid complications.

## Conclusions

Firstly, the early diagnosis of spontaneous rhinoliquorrhea still posses a challenge; any patient presenting with persistent watery discharge from the nose should have the discharge checked for β-trace protein. Imaging like high resolution computed tomography and MRI cisternography may aid in the location of the defect. 

When it comes to the surgical reconstruction of the skull base, the selection of the graft may be up to the surgeons preference; however, closure should be achieved in a combination of overlay and underlay techniques whenever possible. Placement of a lumbar drain does not yield any benefit to the outcome and should therefore be waived. When considering the surgical approach, careful individual planning for each patient is paramount. Due to the considerable recurrence rate over a longer period of time, a close and prolonged follow-up is recommended.

## Abbreviations

- CSF – cerebrospinal fluid
- MRI – magnet resonance imaging

## Notes

### Author contributions

Mark Jakob and Mattis Bertlich contributed equally to this work.

### Competing interests

The authors declare that they have no competing interests.

## Figures and Tables

**Table 1:**
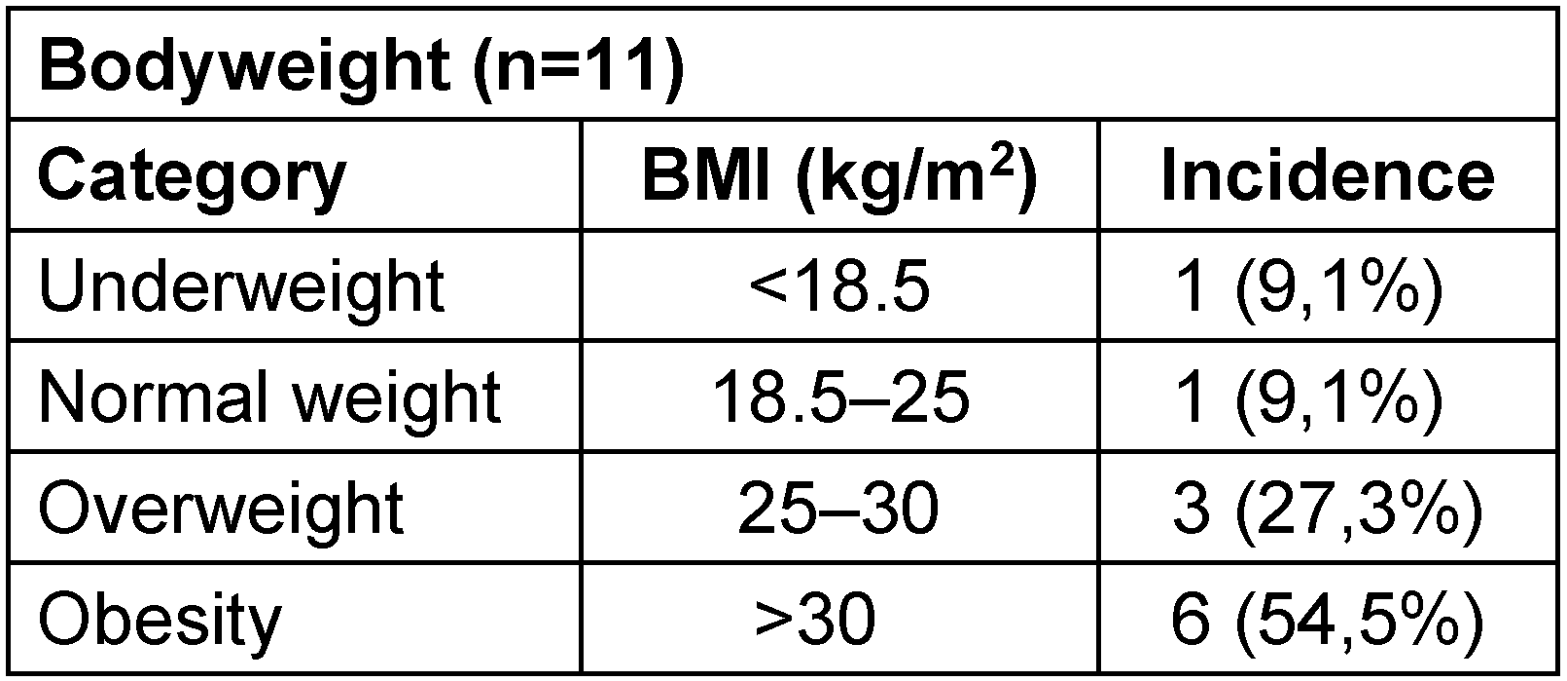
Bodyweight characteristics of patients

**Table 2:**
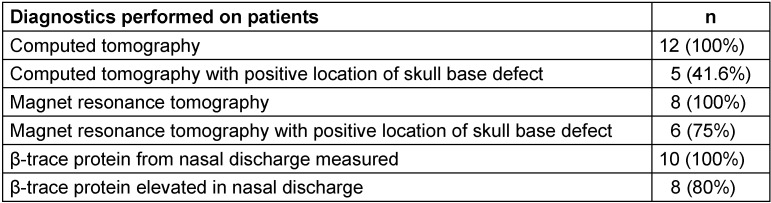
Diagnostic procedures in patients with rhinoliquorrhea

**Table 3:**
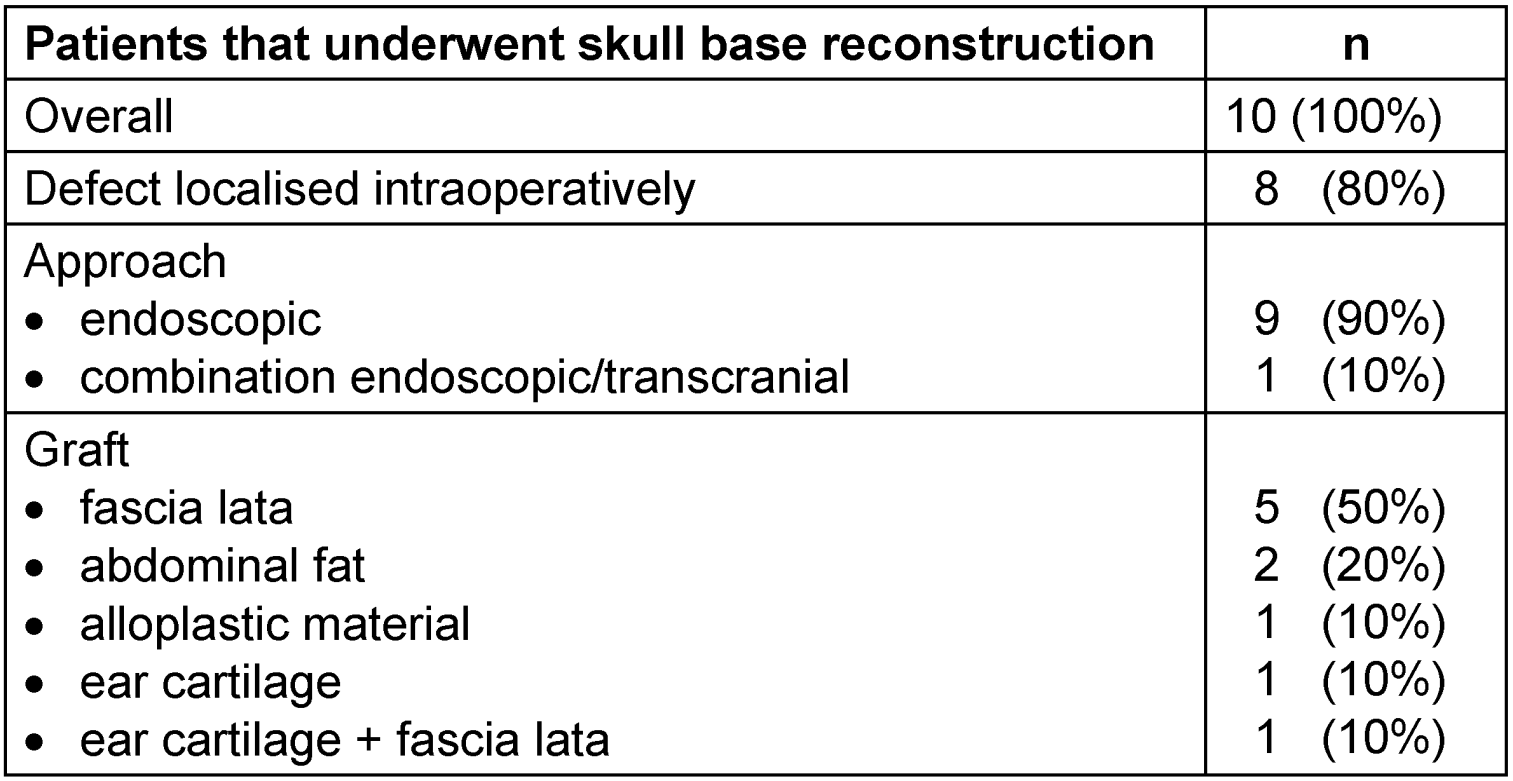
Features of the surgical procedures the patients underwent
